# Chemotherapy-induced tumor immunogenicity is mediated in part by megakaryocyte-erythroid progenitors

**DOI:** 10.1038/s41388-023-02590-0

**Published:** 2023-01-16

**Authors:** Avital Vorontsova, Tim J. Cooper, Jozafina Haj-Shomaly, Madeleine Benguigui, Sapir Levin, Bar Manobla, Rotem Menachem, Michael Timaner, Ziv Raviv, Yuval Shaked

**Affiliations:** 1 Department of Cell Biology and Cancer Science, Rappaport Faculty of Medicine, Technion–Israel institute of Technology, Haifa, Israel; 2 Rappaport Technion Integrated Cancer Center, Haifa, Israel; 3 Department of Immunology, Rappaport Faculty of Medicine, Technion–Israel Institute of Technology, Haifa, Israel; 4 Faculty of Chemical Engineering, Technion - Israel Institute of Technology, Haifa, Israel

**Keywords:** Cancer microenvironment, Tumour immunology

## Abstract

Chemotherapy remains one of the main treatment modalities for cancer. While chemotherapy is mainly known for its ability to kill tumor cells directly, accumulating evidence indicates that it also acts indirectly by enhancing T cell-mediated anti-tumor immunity sometimes through immunogenic cell death. However, the role of immature immune cells in chemotherapy-induced immunomodulation has not been studied. Here, we utilized a mouse pancreatic cancer model to characterize the effects of gemcitabine chemotherapy on immature bone marrow cells in the context of tumor immunogenicity. Single cell RNA sequencing of hematopoietic stem and progenitor cells revealed a 3-fold increase in megakaryocyte-erythroid progenitors (MEPs) in the bone marrow of gemcitabine-treated mice in comparison to untreated control mice. Notably, adoptive transfer of MEPs to pancreatic tumor-bearing mice significantly reduced tumor growth and increased the levels of anti-tumor immune cells in tumors and peripheral blood. Furthermore, MEPs increased the tumor cell killing activity of CD8 + T cells and NK cells, an effect that was dependent on MEP-secreted CCL5 and CXCL16. Collectively, our findings demonstrate that chemotherapy-induced enrichment of MEPs in the bone marrow compartment contributes to anti-tumor immunity.

## Introduction

Pancreatic cancer is the fourth leading cause of cancer related deaths in western countries, with a median survival of several months and a five-year survival of approximately 5%. Treatment options are very limited, mostly involving combinations of chemotherapy such as nab-paclitaxel and gemcitabine [1, 2]. Chemotherapy primarily acts on proliferating cancer cells by inducing DNA damage which then leads to apoptosis [3]. However, its therapeutic activity is also related to immunomodulatory mechanisms [4]. Specifically, following chemotherapy, dying cancer cells release immunostimulatory molecules such as ATP, calreticulin and High Mobility Group Box 1 (HMGB1), collectively known as Damage‐Associated Molecular Patterns (DAMPs). Such DAMPs activate antigen presenting cells (APCs), enhance the production of inflammatory cytokines, and stimulate T cell responses that kill more cancer cells. This process, known as immunogenic cell death (ICD), eventually results in long‐lasting anti-tumor immunity [5].

In contrast to the anti-tumor effect of chemotherapy, several studies have demonstrated the tumor-supporting effects of chemotherapy by different mechanisms. For example, chemotherapy administered at the maximum tolerated dose (MTD) supports the recruitment and activation of immunosuppressive and pro-angiogenic cells [6]. Furthermore, myeloid derived suppressor cells (MDSCs) and monocytes enhance angiogenesis in response to chemotherapy, thereby promoting therapy resistance [6, 7]. In another study, it has been demonstrated that gemcitabine chemotherapy activates the inflammasome in MDSCs, leading to secretion of IL-1β and pro-tumorigenic immune activity [8]. Thus, chemotherapy acts in various ways on the immune cells, altering their composition and activity in the tumor, which eventually affects tumor fate.

Hematopoietic stem and progenitor cells (HSPCs) are the origin of some of the immune cells colonizing tumors. These cells undergo specific differentiation patterns in response to tumor stimuli, leading to immunosuppressive properties [9]. We and others have previously demonstrated that aggressive tumors induce the enrichment of HSPCs in the bone marrow and peripheral blood [9–11]. We showed that HSPCs differentiate into pro-metastatic cells such as myeloid-dendritic progenitors (MDPs), which further differentiate into immunosuppressive macrophages [10]. However, little is known about the effect of chemotherapy on various immune cells in their immature state, and whether such effects dictate a specific pattern of pro- or anti-tumor immunomodulation.

Here we explored the effect of chemotherapy on the composition and activity of HSPCs, and the role of these cells in determining tumor fate. Using single cell RNA sequencing (scRNA-seq) we found that megakaryocyte-erythroid progenitors (MEPs) are enriched in the bone marrow of gemcitabine-treated mice. These cells directly enhance the activity of cytotoxic anti-tumor immune cells, ultimately promoting tumor growth inhibition. Our study thus reveals a new mechanism by which chemotherapy promotes anti-tumor immunity that is independent of immunogenic cell death.

## Results

### MEPs are enriched in the bone marrow of gemcitabine-treated mice

**Fig. 1:**
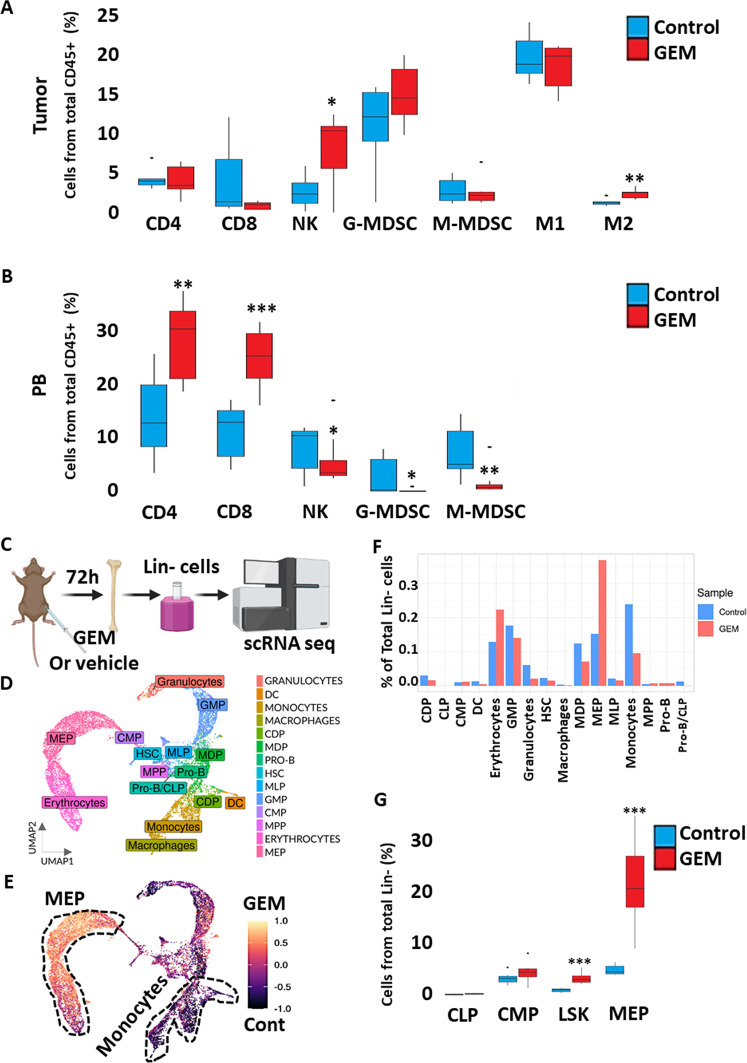
Gemcitabine treatment affects immune cell composition in tumors, peripheral blood and bone marrow. **A**, **B** Panc02 cells (5 × 10^5^ cells/mouse) were orthotopically implanted into the pancreas of C57BL/6 mice (*n* = 7 mice/group). Three weeks later, mice were treated with gemcitabine (GEM, 500 mg/kg) or vehicle control. Tumors and peripheral blood were harvested 72 h post-treatment. The percentages of lymphoid and myeloid cells were assessed in tumors (**A**) and peripheral blood (PB; **B**) by flow cytometry. **C**–**G** Naïve, tumor-free mice were treated with GEM or vehicle control and sacrificed 72 h later. **C** Lin- cells were obtained from the bone marrow and analyzed by single cell RNA sequencing (scRNA-seq) and flow cytometry. A schematic representation of the experimental plan is shown. **D** UMAP plot of hematopoietic stem and progenitor cells (HSPCs). **E** HSPC abundance was determined by the scRNA-seq; gemcitabine (GEM) vs. control groups. **F** HSPCs subsets were quantified and are shown as percentages of total Lin- cells. **G** HSPCs were quantified by flow cytometry. Statistical significance was assessed by unpaired one-tailed t-test. Significant *p*-values are shown as *p < 0.05, **p < 0.01, ***p < 0.001.

To study the effect of gemcitabine chemotherapy on immune cell composition in peripheral blood and tumors, we utilized an orthotopic pancreatic cancer mouse model in which the Panc02 cell line was implanted in the pancreas. When tumors reached 50 mm^3^, mice were treated with gemcitabine administered at the MTD and the levels of lymphoid and myeloid cells in peripheral blood were analyzed after 24 h, 72 h and one week. A significant increase in the lymphoid to myeloid ratio was observed at the 72 h time point (Fig. S1). At this time point, the composition of different immune cells at the tumor and peripheral blood displayed changes in lymphoid and myeloid cells. For example, CD8 + and CD4 + T cells are increased in peripheral blood whereas NK cells are increased in tumors. In addition, MDSC levels (both granulocytic and monocytic MDSCs) were decreased in peripheral blood, further indicating that the chemotherapy modulates the immune system mostly towards anti-tumor immune activity (Fig. 1A, B, and Fig. S2A, B). Furthermore, Granzyme B expression was increased in tumors derived from gemcitabine-treated mice in comparison to control tumors, suggesting that gemcitabine treatment increases cytotoxic immune activity at this time point (Fig. S2C). Importantly, similar anti-tumor immune cell composition was observed in mice bearing Panc02 tumors that received long-term treatment consisting of three weekly cycles of gemcitabine (Fig. S3), further indicating that continuous administration of gemcitabine maintains an anti-tumor immune effect.

The role of immature cells and specifically HSPCs has been studied in the context of cancer [9–11]. However, the effect of gemcitabine therapy on these cells has not been fully explored. To investigate this, we used scRNA-seq to analyze bone marrow cells extracted from tumor-free mice 72 h after treatment with gemcitabine or vehicle control (Fig. 1C). MEPs were highly enriched (~3-fold) in bone marrow derived from the gemcitabine-treated mice compared with control (Fig. 1D–F and Fig. S4). Similar enrichment of MEPs was found in the bone marrow of mice bearing Panc02 tumors treated with a single dose or three weekly cycles of gemcitabine, as assessed by flow cytometry (Fig. 1G and Fig. S5, respectively). Overall, these results demonstrate that gemcitabine treatment induces an enrichment of MEPs in the bone marrow compartment.

### Characterization of MEPs in gemcitabine-treated mice

**Fig. 2:**
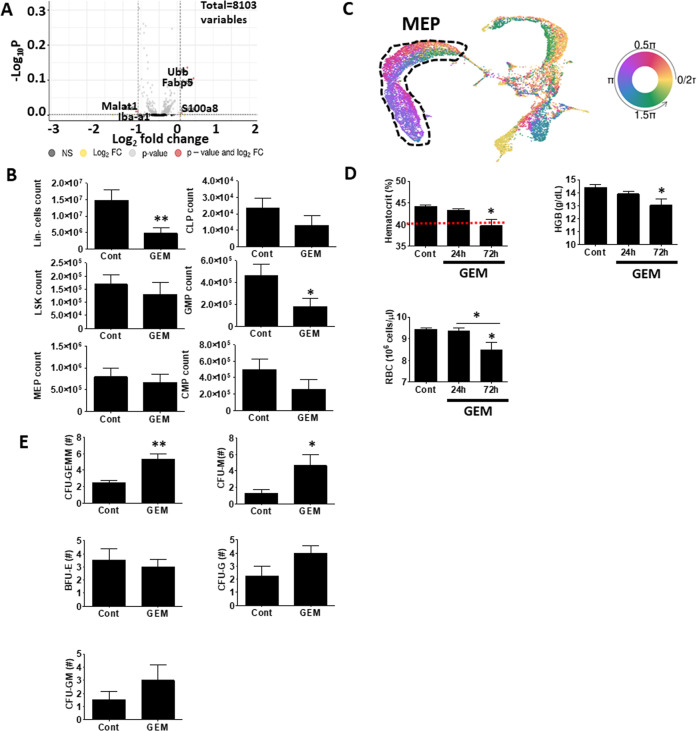
Gemcitabine treatment decreases the level of Lin- cells in the bone marrow and increases proliferative potential of megakaryocyte-erythroid progenitors. **A**–**C** Tumor-free mice were treated with gemcitabine (GEM) or vehicle control and sacrificed 72 h later. Bone marrow was harvested and analyzed by single cell RNA sequencing or flow cytometry. Differentially expressed genes in megakaryocyte-erythroid progenitors obtained from gemcitabine-treated and control mice are presented in a volcano plot (**A**). **B** Absolute numbers of the indicated cell types as assessed by flow cytometry are shown. **C** UMAP plot of hematopoietic stem and progenitor cells (HSPCs) representing the expression of cell cycle genes. 0.5 π indicates initial S phase, π indicates initial G2M phase, 1.5 π indicates middle of M phase and 1.75–0.25 π indicates G1/G0 state. **D** Tumor-free C57BL/6 mice were treated with gemcitabine (GEM) or control (*n* = 4 mice/group) and blood was drawn 24 h and 72 h later. Hematocrit percentage, red blood cell (RBC) count, and hemoglobin (HGB) concentration were determined. Red dashed line indicates the lowest physiological level of hematocrit. **E** A MethoCult assay was performed using Lin- cells extracted from bone marrow of gemcitabine-treated (GEM) or control mice. The indicated colony types were counted. Statistical significance was assessed by one-way ANOVA, followed by Tukey post-hoc test when comparing more than two groups or unpaired one-tailed *t*-test when comparing two groups. Asterisks represent significance from control, unless indicated otherwise. Significant *p*-values are shown as *p < 0.05; **p < 0.01.

We next asked whether the enriched MEP population displays different functional characteristics following therapy. ScRNA-seq analysis demonstrated relatively similar gene expression patterns in MEPs derived from the bone marrow of control and gemcitabine-treated mice suggesting similar functional characteristics (Fig. 2A). Since chemotherapy results in myelosuppression [12], we also asked whether the enrichment of MEPs is due to a reduction in the levels of different cell types, enhanced MEP proliferation in response to gemcitabine therapy, or both. To test this, we analyzed the absolute number of different Lin- cells in the bone marrow compartment of mice treated with gemcitabine or vehicle control. While the absolute number of MEPs was similar in the bone marrow of gemcitabine-treated and control mice, reduced numbers of Lin- cells (among them, GMPs, CLPs, and CMPs) were detected in the bone marrow of gemcitabine-treated mice (Fig. 2B). These results suggest that gemcitabine depletes the number of different immune cell populations, which in turn, may explain the increased proportion of MEPs in the bone marrow.

We next assessed whether gemcitabine affects MEPs proliferative and/or differentiation capacity. Gene expression data from the scRNA-seq analysis revealed expression of genes associated with cell proliferation, suggesting that MEPs can potentially be more proliferative, although it was not seen at the 72 h time point (Fig. 2C). Supporting evidence for the potential enrichment of MEPs in gemcitabine-treated mice was shown by reduced hematocrit, RBCs and hemoglobin in the blood of treated mice compared to control. These results further support that the enrichment in MEPs arises in response to the lack of red blood cell components, which may indicate a potential compensatory process (Fig. 2D). In addition, in a MethoCult assay, we found that CFU-GEMM and CFU-M colonies were significantly elevated in the bone marrow of mice treated with gemcitabine compared to control mice (Fig. 2E). CFU-GEMM colonies serve, in part, as a readout for MEP enrichment [13, 14].

Further characterization of MEPs revealed that they uniquely express ERMAP when compared to all other Lin- cells, as analyzed by scRNA-seq and validated by flow cytometry, therefore providing an additional tool for their isolation (Fig. S6). Of note, a bioinformatic analysis of differentially expressed genes between MEPs and all other Lin- cells revealed an enrichment of pathways associated with platelets and red blood cells (RBC) further supporting that the cluster of cells is indeed associated with MEPs (Fig. S7). Taking the aforementioned experiments together, these results suggest that gemcitabine contributes to MEP enrichment primarily by the depletion of different Lin-cell populations at the 72 h time point. In addition, it may also enrich MEPs by other pathways, some of which are likely associated with a feedback loop compensating for chemotherapy-induced thrombocytopenia and anemia [15], although these pathways were not directly assessed in these experimental settings.

### MEPs promote anti-tumor immune activity

**Fig. 3:**
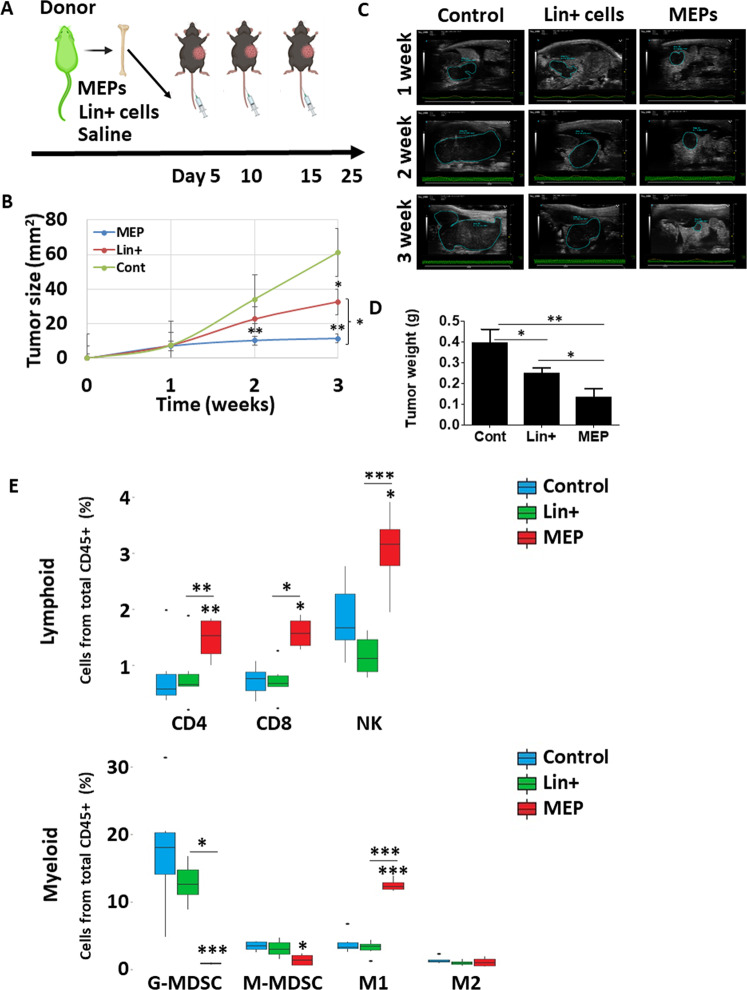
Adoptive transfer of megakaryocyte-erythroid progenitors enhances anti-tumor activity. **A** Megakaryocyte-erythroid progenitors (MEPs) or Lin+ cells were isolated from bone marrow of GFP + mice. The GFP-tagged MEPs or Lin+ cells (5 × 10^5^ cells/mouse) were injected every 5 days to mice bearing Panc02 tumors (*n* = 4–6 mice/group). Control mice were injected with saline. A schematic representation of the adoptive transfer experiment is shown. **B**–**C** Tumor growth was assessed by micro-ultrasound (US) imaging. Tumor size was plotted (**B**). **C** Representative US images are shown. **D** When control tumors reached endpoint, mice were sacrificed, tumors were removed and subsequently weighed. **E** The tumors were prepared as single cell suspensions and the levels of different lymphoid and myeloid cells were assessed by flow cytometry. Statistical significance was assessed by one-way ANOVA, followed by Tukey post-hoc test. Asterisks represent significance from control, unless indicated otherwise. Significant *p*-values are shown as *p < 0.05; **p < 0.01; ***p < 0.001.

We next asked whether MEP enrichment affects tumor immunogenicity. To this end, we adoptively transferred GFP-tagged MEPs derived from the bone marrow of tumor-free donor mice to recipient mice bearing Panc02 tumors. As controls, Panc02 tumor-bearing mice received either saline or Lin+ cells (Fig. 3A). Tumor growth was assessed regularly using micro ultrasound (US). When the saline control group reached endpoint, tumors were removed and assessed for weight and composition of different immune cells. A significant reduction in tumor growth was observed in mice that received MEPs in comparison to the saline control group. Reduced tumor growth was also observed in the Lin+ control group, although to a lesser extent (Fig. 3B–D). Importantly, the levels of NK cells, CD8 + T cells, and pro-inflammatory macrophages were significantly increased while immunosuppressive macrophages were significantly decreased in tumors of mice adoptively transferred with MEPs compared to control or Lin+ groups (Figs. 3E and S8A). No changes in the levels of lymphoid and myeloid cells were detected in peripheral blood (Fig. S8B). Of note, the adoptively transferred GFP + cells were detected in the bone marrow of recipient mice, and the percentage of MEPs was elevated in the MEPs adoptively transferred group compared to all other groups (Fig. S9). These results confirm that MEPs home to their natural site in the bone marrow, and potentially support anti-tumor immunity.

In a parallel adoptive transfer experiment, we extended the experimental timeline to allow the tumors in the MEP adoptive transfer group to reach their own endpoint (Fig. S10A). Mice adoptively transferred with MEPs survived 2 weeks longer than control mice receiving saline (Fig. S10B). In addition, tumors from mice adoptively transferred with MEPs displayed significantly higher levels of NK cells and pro-inflammatory macrophages, lower levels of immunosuppressive macrophages and increased Granzyme B expression (Fig. S10C–E). Taken together, these findings demonstrate that MEPs contribute to anti-tumor immunity, even without the use of gemcitabine chemotherapy.

### MEPs directly contribute to cytotoxic immune cell activity

**Fig. 4:**
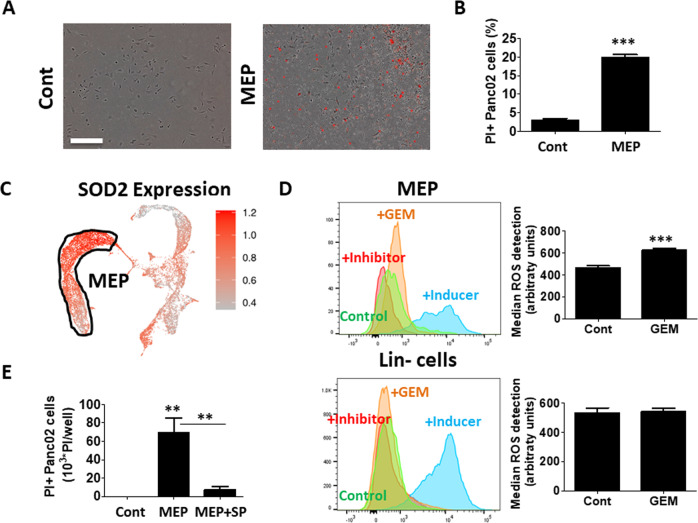
Megakaryocyte-erythroid progenitors directly directly induce tumor cell killing. **A**, **B** Panc02 cells were cultured for 24 h with megakaryocyte-erythroid progenitors (MEPs; 2.5 × 10^5^/ml). Representative IncuCyte images are shown. Scale bar = 300 μm (**A**). The percentage of propidium iodide (PI) positive tumor cells (representing dead cells) was analyzed by flow cytometry (**B**). **C** SOD2 mRNA expression level in MEPs was determined from the scRNA-seq dataset. Shown is the UMAP plot of the total data. **D** Panc02 tumor-bearing C57BL/6 mice (*n* = 7 mice/group) were treated with gemcitabine (GEM) or vehicle control. After 72 h, bone marrow cells were harvested and Lin- cells and MEPs were isolated. ROS activity was determined. Left panel represents flow cytometry histograms, and right panel represents the average of median ROS detection. **E** Panc02 cells, cultured with MEPs as in **A**, **B**, were also cultured in the presence or absence of sodium pyruvate (SP) as a ROS scavenger. The percentage of PI-positive tumor cells (representing dead cells) was analyzed as in **B**. Statistical significance was assessed either by one-way ANOVA followed by Tukey post-hoc test, when comparing more than two groups, or by unpaired two-tailed t-test, when comparing two groups. Significant *p*-values are shown as **p < 0.01; ***p < 0.001.

Our findings demonstrating MEP-induced anti-tumor immunity prompted us to explore whether direct communication exists between MEPs and cytotoxic immune cells. We first analyzed whether MEPs, on their own, have anti-tumor activity, and found that they can directly increase Panc02 tumor cell killing (Fig. 4A, B). Previous studies demonstrated that reactive oxygen species (ROS) are upregulated following megakaryocyte development and differentiation [16]. We therefore asked whether ROS is increased in MEPs, which could explain their direct anti-tumor activity. To test this possibility, we analyzed the scRNA-seq dataset for the expression of enzymes associated with ROS. We found that SOD2 is highly expressed in the MEP population (Fig. 4C). Next, ROS activity was assessed in MEPs and Lin- cells obtained from gemcitabine-treated and control mice. MEPs from gemcitabine-treated mice displayed increased ROS activity compared with MEPs from control mice, whereas ROS activity in Lin- cells remained unchanged (Fig. 4D). Indeed, the addition of ROS scavenger to MEPs resulted in a reduced tumor cell killing effect (Fig. 4E). While these results suggest that MEPs directly promote tumor cell killing, the fact that MEPs are located at the bone marrow compartment, distant from the tumor site, further implies that ROS, a short-lived molecule, may not play a role in our experimental setting.

**Fig. 5:**
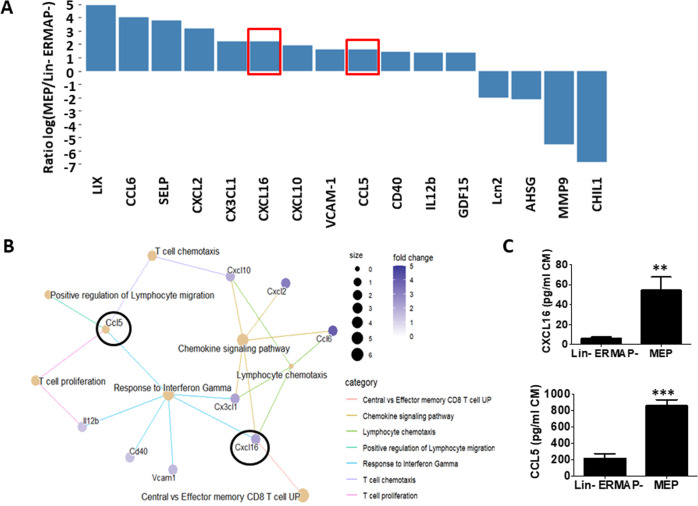
MEPs promote cytotoxic immune cell activity by secreted factors. **A** Conditioned medium from megakaryocyte-erythroid progenitor (MEP) cultures and MEP-depleted Lin-cell cultures were applied to a cytokine and chemokine array. The levels of selected proteins are shown as log ratios (MEPs vs. MEP-depleted Lin- cells, Lin-ERMAP- cells). Proteins known to be associated with CD8 + T cell and NK cell activity are indicated with a red box. **B** Differentially expressed genes were assessed by GO to identify associated biological pathways. CXCL16 and CCL5 (circled in black) were displayed as hubs. **C** CXCL16 and CCL5 levels were analyzed by specific ELISA in conditioned medium of MEPs vs. MEP-depleted Lin- cells. Statistical significance was assessed by unpaired two-tailed t-test. Significant p values are shown as **p < 0.01; ***p < 0.001.

Next, we analyzed the conditioned medium of MEPs, reasoning that MEPs exert their anti-tumor immunity via secreted factors. To this end, we compared the secretomes of MEPs and of MEP-depleted Lin- cells using a protein array. We found that the MEP-conditioned medium contained higher levels of several factors, some of which are known to be involved in lymphocyte chemotaxis, effector CD8 + T cells, positive regulation of lymphocyte migration, and T cell proliferation (Fig. 5A, B). Among the identified MEP-secreted factors we note the presence of CXCL16 and CCL5, which are known to activate anti-tumor immune cells such as CD8 + T cells and NK cells in different tumor models [17, 18]. We validated the increased levels of these factors in MEPs compared with MEP-depleted Lin- cells using ELISA (Fig. 5C).

**Fig. 6:**
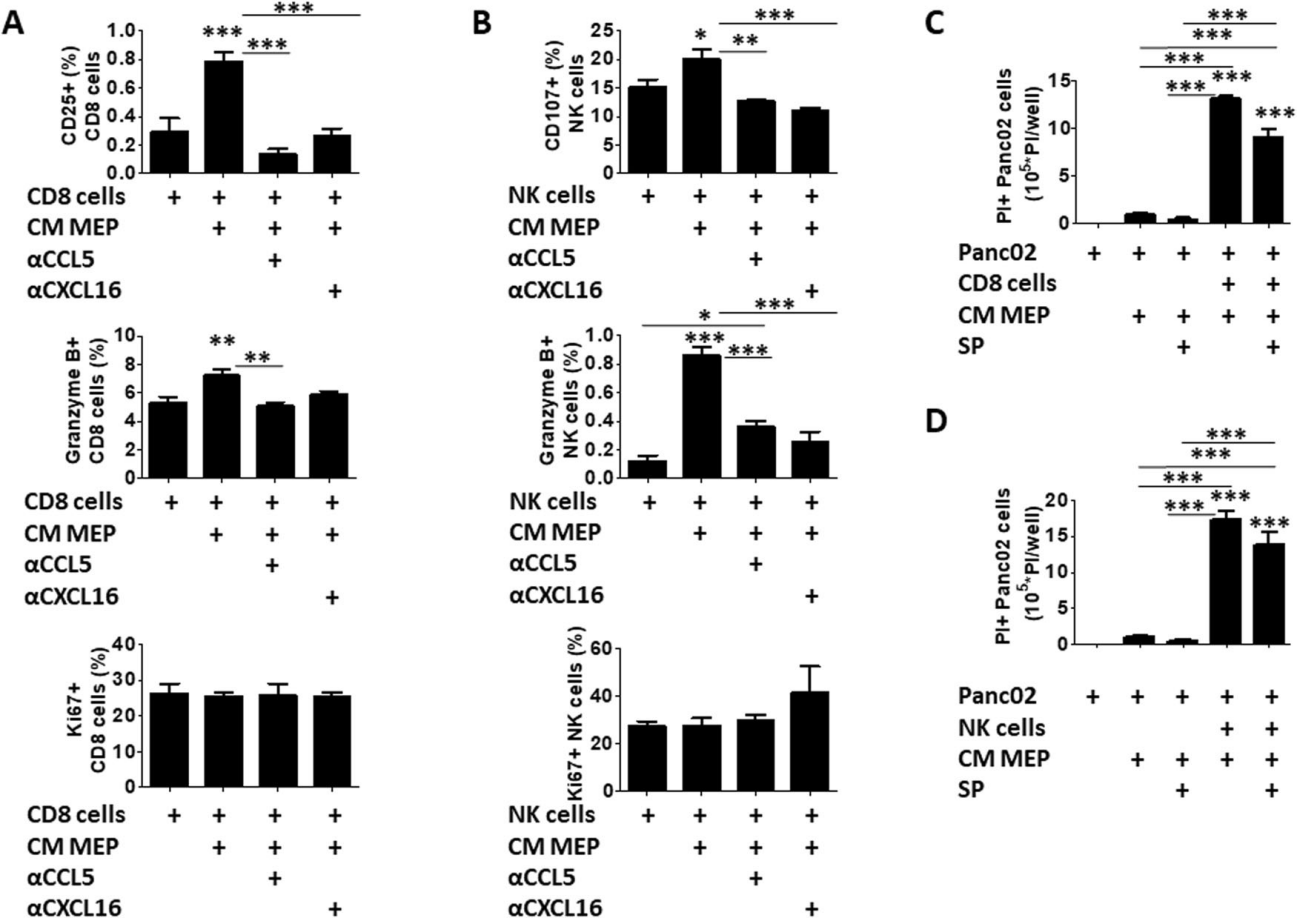
Increased cytotoxic immune cell activity by MEPs is mediated in part by CXCL16 and CCL5. **A**, **B** CD8 + T cells (**A**) and NK cells (**B**) were cultured in the presence of conditioned medium (CM) obtained from MEP cultures, or MEP CM depleted of CCL5 or CXCL16. Cell activity, Granzyme B expression and cell proliferation were analyzed by flow cytometry. **C**, **D** Panc02 cells were cultured for 24 h with CD8 + T cells (**C**) or NK cells (**D**) in the presence of MEP-conditioned medium as well as a ROS scavenger, sodium pyruvate (SP). The percentage of PI-positive tumor cells (representing dead cells) was analyzed by flow cytometry. Statistical significance was assessed by one-way ANOVA followed by Tukey post-hoc test. Asterisks represent significance from control, unless indicated otherwise. Significant p values are shown as *p < 0.05; **p < 0.01; ***p < 0.001.

To determine the effect of MEP-secreted CXCL16 and CCL5 on the activity of CD8 + T cells and NK cells, we analyzed the cytotoxic activity of these cells in vitro in the presence of MEP-conditioned medium that was depleted of CXCL16 or CCL5, respectively. As expected, MEP-conditioned medium activated CD8 + T cells and NK cells, but conditioned medium depleted of CXCL16 or CCL5 failed to activate CD8 + T cells and NK cells, while proliferation rate of these cells was unchanged (Fig. 6A, B). Note that the use of conditioned medium rather than co-culture systems of MEPs and T cells or NK cells rules out the possibility of ROS-induced anti-tumor immunity, as was also evaluated when using ROS scavenger (Fig. 6C, D).

Next, since CCL5 and CXCL16 secreted from MEPs located at the bone marrow niche may affect immune cells at the tumor microenvironment, we measured the levels of CXCL16 and CCL5 in peripheral blood and tumors, 72 h after treatment with gemcitabine chemotherapy to see whether they can explain the anti-tumor immunity. Both CXCL16 and CCL5 demonstrated increased levels in peripheral blood and tumors following treatment with gemcitabine, although some of the results did not reach statistical significance (Fig. S11A, B). These results were also observed in tumors treated with three weekly cycles of gemcitabine (Fig. S11C). Taken together, these findings suggest that following gemcitabine therapy, MEPs directly affect CD8 + T cells and NK cells probably via the secretion of CXCL16 and CCL5, thereby contributing to anti-tumor immunity.

## Discussion

The anti-tumor activity of chemotherapy is primarily associated with its ability to kill rapidly dividing cancer cells [19]. Recent studies have revealed ICD as an additional mechanism which explains tumor cell killing effect [20]. However, chemotherapy may also induce mechanisms that negate its anti-tumor activity, specifically via immunosuppression and associated lymphopenia and thrombocytopenia [12, 21]. In our study, we describe an immunomodulatory mechanism by which gemcitabine chemotherapy enhances the tumor cell-killing effect. We demonstrate that gemcitabine reduces the levels of immature bone marrow derived cells, resulting in an enrichment in MEPs, a subpopulation of HSPCs. Such MEPs contribute to anti-tumor immunity by directly activating cytotoxic T cells and NK cells. Evidently, when Panc02 tumor bearing mice were adoptively transferred with MEPs, tumor growth was remarkably slowed down, due in part to the enrichment of cytotoxic immune cells within tumors. We further demonstrated that these effects are attributed, at least in part, to the increased expression of CXCL16 and CCL5 by MEPs, which in turn support the anti-tumor activity of CD8 + T cells and NK cells. Indeed, CXCL16 and CCL5 have already been shown to be associated with increased activity and infiltration of T cells and NK cells to different sites, further supporting the notion that these molecules directly affect anti-tumor immunity [22–25]. Interestingly, in addition to the direct effect of MEPs on anti-tumor immunity, we also found that, following gemcitabine therapy, MEPs generated higher ROS levels. This molecule can explain the direct tumor cell killing effect reported in co-culture systems. Nevertheless, it should be noted that the fact that MEPs are located in the bone marrow compartment, distant from the tumor site, further suggests that cytokine secretion rather than ROS activity supports the anti-tumor activity. Taken together, our study is the first to demonstrate chemotherapy-induced enrichment of cell populations that in turn activate anti-tumor cytotoxic cells.

The effect of gemcitabine in pancreatic cancer has been evaluated both in mice and patients. It has been shown that the absolute count of T cells is significantly reduced within the first few days after gemcitabine administration; however, their anti-tumor activity, as measured by INF-γ, increases [26]. In support of this, here we show increased cytotoxic activity in response to gemcitabine chemotherapy, as demonstrated by an increase in Granzyme B. We also show that gemcitabine induces anemia in the form of reduced levels of hematocrit, hemoglobin and RBCs. These results are in line with clinical studies demonstrating that gemcitabine chemotherapy causes mild to moderate anemia in cancer patients [27]. This anemia can also explain the enrichment of MEPs, as they are immature cells that can differentiate into RBCs and platelets. Indeed, while we show that MEPs are enriched as a result of the suppression of other immature immune cells (such as CLP and GMP), our scRNA-seq analysis revealed that MEPs display greater proliferative potential than all other Lin- cells. This suggests that in addition to the depletion of different cell populations in the bone marrow following chemotherapy, MEP can potentially be enriched due to a feedback loop in response to chemotherapy-induced anemia. We assume that MEPs further differentiate into erythroid progenitors, as previously shown [13], compensating for the anemia caused by gemcitabine chemotherapy.

The differentiation of MEPs from HSCs is induced by GATA-1, a zinc finger transcription factor [28]. A previously published scRNA-seq dataset revealed that MEPs are in a transition state comprising both common myeloid progenitor (CMP) as well as megakaryocyte and erythrocyte linages [13]. In agreement, our scRNA-seq analysis revealed that MEPs are positioned between CMPs and megakaryocytes and/or erythrocytes. Both cell extrinsic and intrinsic factors affect MEP fate. Among these factors are erythropoietin, thrombopoietin and stem cell factor (SCF), all of which play a significant role in the maturation of MEPs into either erythrocytes or megakaryocytes [29]. To further support these findings, using the MethoCult assay, we demonstrate that CFU-GEMM colonies are enriched in Lin- cells obtained from gemcitabine-treated mice compared to control mice. These colonies can further differentiate into megakaryocyte progenitors and erythroid progenitors [13]. Collectively, these results indicate that gemcitabine can induce the enrichment of MEPs.

Our previous studies demonstrate that in addition to the therapeutic activity of anticancer drugs, among them chemotherapy, the host generates pro-tumorigenic activities which in turn contribute to tumor re-growth and resistance to therapy [30, 31]. We previously showed that unlike paclitaxel, gemcitabine has little effect on the invasiveness of tumor cells following therapy [30]. However, in another study of pancreatic cancer, we reported increased metastatic lesions in gastrointestinal organs following treatment with MTD gemcitabine [32]. Here, we did not observe increased metastasis in gemcitabine-treated mice. We note, however, that the majority of our experiments were performed in a short-term format, wherein mice were sacrificed three days after a single treatment with gemcitabine, and our study did not focus on the metastatic sites of pancreatic cancer. Importantly, MEP enrichment following therapy was also observed after three weekly cycles of gemcitabine therapy, further supporting its potential for long-term anti-tumorigenic immunity. However, the potential role of gemcitabine-induced anti-tumor immunity on metastasis formation requires additional studies.

In summary, our study highlights an additional mechanism by which chemotherapy exerts a therapeutic effect. We show, for the first time, that chemotherapy-induced MEP enrichment contributes to anti-tumor immunity. This study may also explain the synergistic therapeutic effect observed when chemotherapy is combined with immunotherapy [33]. Overall, our study sheds light on the effects of chemotherapy on immature immune cells which in turn affect tumor fate.

## Materials and methods

### Cell culture

Murine Panc02 pancreatic adenocarcinoma cell line was obtained from the American Type Culture Collection (ATCC, Manassas, VA). The cells were thawed from original stocks and used within 4 months of resuscitation. Panc02 cells were cultured in DMEM supplemented with 10% fetal bovine serum. The medium contained 1% L-glutamine, sodium pyruvate and penicillin–streptomycin. The cells were routinely tested to be mycoplasma free.

### Tumor models and treatment

All animal work was approved by the Technion Institutional Animal Care and Use Committee and performed according to the National Institutes of Health guidelines and relevant ethical regulations. Eight-ten week old female C57BL/6 mice (Envigo, Israel) were used in the animal experiments. Mice were age-matched and randomized per experiment. The groups were blinded to the person who routinely assessed them. More than 4 mice per group were used to reach statistical significance.

For orthotopic tumor models, Panc02 cells (0.5 × 10^6^ per mouse) were mixed in serum-free DMEM medium and were injected (in a volume of 15 μL) trans-peritoneally into the head of the pancreas of mice, as previously described [32]. Tumor size was assessed once a week using micro ultra-sound (VisualSonics Vevo® 3100 Imaging System). Gemcitabine was administered as previously described at a dose of 500 mg/kg mouse [6].

### Isolation of lineage-negative cells and megakaryocyte-erythroid progenitors from bone marrow

Female C57BL/6 or GFP mice (8–10 weeks of age) were sacrificed, and femurs and tibiae were flushed with sterile PBS to obtain bone marrow. Subsequently, bone marrow cells were filtered through a 70 µm pore size cell strainer (BD Biosciences, Bedford, MA). Red blood cells (RBC) were lysed using a sterile lysis buffer (8.26 g/L ammonium chloride, 1 g/L sodium bicarbonate and 0.01 M EDTA). Lineage-positive (Lin + ) cells were isolated by magnetic-activated cell sorting beads (MACS) using the MagCellect mouse hematopoietic cell lineage depletion kit in accordance with the manufacturer’s protocol (R&D Systems, Minneapolis, MN)s. The Lin+ cells were stored and used separately. MEPs were isolated from the remaining Lin- cells using erythroid membrane-associated protein (ERMAP)-biotin antibody (Bioss antibodies, bs-12333R-Biotin). ERMAP-positive cells were collected and re-suspended in sterile PBS. In some experiments, Lin- cells depleted from MEPs were used. The depletion was carried out by magnetic beads conjugated with ERMAP biotinylated antibody. The cell populations were used for different purposes as described below.

### Adoptive transfer

MEPs or Lin+ cells were isolated from bone marrow of GFP + donor mice. The GFP-tagged MEPs or Lin+ cells (5 × 10^5^ cells/mouse) were intravenously injected to recipient mice bearing Panc02 tumors (*n* = 4–7 mice group). Control mice were injected with saline. Adoptive transfer was performed 3 times every 5 days, starting on day 5 after orthotopic implantation of Panc02 cells. Reconstitution recovery of GFP + donor cells was analyzed after 3–5 weeks in bone marrow, peripheral blood and tumors by flow cytometry.

### Conditioned medium preparation

MEPs or Lin- cells depleted of MEPs (1 × 10^6^ cells/ml) were cultured in DMEM serum-free medium for 24 hours to generate conditioned medium (CM). CM (1 ml) was depleted of CCL5 or CXCL16 by overnight incubation at 4 °C with 1 µg anti-CCL5 (AF478, R&D Systems) or anti-CXCL16 (AF503, R&D Systems) antibodies, respectively. The CM was then incubated with 50 µl Protein G Sepharose® beads (ab193259, Abcam) for 1 h at 4 °C. Beads were removed by centrifugation in 2000 g for 10 min.

### Tumor lysate preparation and protein measurement

Panc02 tumors were extracted from sacrificed mice. Tumor tissue was placed in a 1.5 mL tube containing RIPA buffer (5 M NaCl, 0.5 M EDTA pH = 8, 1 M Tris pH = 8, 1% NP-40, 10% sodium deoxycholate, 10% SDS) and protease inhibitor cocktail (1:100, Sigma-Aldrich, St Louis, Missouri, USA). Stainless steel beads (SSB14B, Next Advance, New York, USA) were added and tumor tissue was homogenized using the Bullet Blender Tissue Homogenizer (Next Advance, Troy, NY) according to the manufacturer’s protocol. The homogenate was centrifuged and supernatant was collected. The protein concentration of the tumor lysates was determined using Protein Assay Dye Reagent Concentrate (Bio-Rad, California, USA). The quantification of Granzyme B, CXCL16 and CCL5 was carried out by a specific enzyme-linked immunosorbent assay (ELISA) kit (R&D Systems) in accordance with the manufacturers’ instructions. All experiments were performed using at least three biological repeats.

### Cytokine arrays and biological pathway enrichment analysis

Panc02 tumor-bearing mice were treated with gemcitabine or vehicle control and sacrificed 72 h later. Peripheral blood was harvested and plasma was isolated by using EDTA tubes. Plasma samples, or CM from MEPs or MEP-depleted Lin- cells, were applied to a proteome profiler mouse XL cytokine array (ARY028, R&D Systems) in accordance with the manufacturer’s instruction. Relative levels of the different proteins were calculated based on densitometry and presented as a log ratio between gemcitabine and control plasma, or between MEP and MEP-depleted Lin- CM. In the CM experiment, upregulated proteins with log Fold Change >2 were used in a biological pathway enrichment analysis. Gene Ontology (GO) category biological processes were determined using the R package *clusterProfiler* [v4.2.2.] [34]. Subsequently, network-based representations were generated from the chosen GO terms using the ‘cnetplot’ function of the *clusterProfiler* package.

### Evaluation of cytotoxic cell activity and tumor cell killing

CD8 + T cells and NK cells were isolated from the spleens of Panc02 tumor-bearing mice using negative selection MojoSort™ Mouse CD8 + T Cell Isolation Kit (BioLegend, Sand Diego, CA), and positive selection NKp46-biotin antibodies (BioLegend), respectively. For immune cell activation, 50,000 CD8 + T cells or NK cells were cultured in full DMEM medium for 96 h at 37 °C in the presence or absence of 25,000 MEPs (ratio of 2:1), or in the presence of 50 μL MEP CM (reaching 1:1 volume ratio). Subsequently, cells were centrifuged at 470 × g for 5 min at room temperature. Cell pellets were re-suspended in recommended medium (2% fetal calf serum, 0.1 mM EDTA in PBS), and the levels of total CD8 + T cells, NK cells and activated cells (CD8 + CD25 + or NKp46 + CD107 + ) were quantified by flow cytometry. In parallel, the cells, as above, were permeabilized, fixed and evaluated for granzyme B expression by flow cytometry using FITC anti-human/mouse granzyme B recombinant antibody (BioLegend), in accordance with the manufacturer’s instructions.

For the Panc02 tumor killing assay, CD8 + T cells and NK cells (5 × 10^5^/ml) were co-cultured with Panc02 cells (4 × 10^4^/ml) in full DMEM medium for 24 h at 37 °C in the presence or absence of MEPs (25 × 10^4^/ml) or MEP CM (1:1 ratio with full medium). Propidium iodide (PI, 500 nM) was added to cultures in order to identify dead cells. T-cell killing effect was monitored using Incucyte Zoom HD/2CLR system (Essen BioScience, Ann Arbor, MI) and dead cells were quantified by flow cytometry. All experiments were performed in at least five biological replicates.

### Flow-cytometry acquisition and analysis

Single-cell suspensions prepared from tumors, bone marrow or peripheral blood cells as previously described [35], were immunostained with antibodies purchased from BioLegend (BLG, San Diego, CA) or BD Biosciences (BD, Franklin Lakes, NJ) in accordance with the manufacturers’ instruction. Bone marrow cells were immunostained with lineage cocktail (17A2/RB6-8C5/RA3-6B2/Ter 119/M1/70)-BV421, Sca1(D7)-BV786, CD117(2B8)-APC, CD34(HM34)-PE, IL-7R(A7R34)-BV605, FCγR(93)-BV510 and ERMAP-AF594 (bs-12333R-A594,Bioss). Tumor cells and peripheral blood cells were immunostained for CD45(30-F11)-AF700 or FITC,F4/80(BM8)-PE, CD11b(M1/70)-PerCP, CD206(C068C2)-BV421, CD11c(N418)-APC-Cy7, Ly6C(1A8)-BV605, Ly6G(HK1.4)-BV510 or for CD45(30-F11)-AF700 or FITC, CD3ε(30-F11)-Alexa Fluor 700, CD8a(53-6.7)-APC/cy7, CD4(GK1.5)-BV510, CD107(1D4B)-BV421, CD25(PC61)-APC, B220(RA3-6B2)-BV605, NKp46(29A1.4)-PE/Cy7, Ki67(16A8)-PE. All antibody mixtures used to identify the different cell populations are indicated in Table S1. For assessing total number of cells in bone marrow, DragonGreen 7.32 µm counting beads (FSDG007, Bangs Laboratories) were diluted 1:50 with PBS. Ten µl (13,635 beads) were added to each tube containing cells flushed from one femur bone. Events were acquired and bead numbers were obtained. At least 100,000 events were acquired using the LSRFortessa flow analyzer system (BD Bioscience) followed by analysis using the FlowJo 10.2 software (Ashland, OR).

### ROS activity assay

ROS activity was determined using a ROS detection kit (Enzo Life Sciences, NY) in accordance with the manufacturer’s instructions. Briefly, bone marrow cells from gemcitabine-treated or control mice were extracted as described above. Subsequently, cells were washed and resuspended in ROS detection solution. In some experiment, sodium pyruvate (10 nM) was used as a ROS scavenger in cultured cells. As a positive control, cells were treated with apocynin. The cells were immunostained with lineage cocktail to analyze the different cell populations. ROS activity was detected in the FITC channel as acquired by flow cytometry.

### Single cell RNA-sequencing

Naïve female C57BL/6 mice (8–10 weeks of age) were treated with gemcitabine or vehicle control. After 72 h, bone marrow cells were harvested and Lin- cells were isolated. The cells were then washed in PBS containing 0.04% BSA and re-suspended at a concentration of 1000 cells/μl in PBS. RNA was extracted and immediately acquired by the 10X Genomics single cell sequencing system, following the manufacturer’s instruction. Bioinformatic analysis was then carried out as described below.

### Single cell RNA-seq alignment and pre-processing

Raw, Illumina base calls (BCLs) were demultiplexed and the resulting FASTQ files were aligned to the mm10 (GRCm38, Ensembl 93) murine reference genome using *10x Genomics CellRanger* [v 5.0.1] to generate expression matrices. 83.7–84.7% of reads mapped to the transcriptome across all samples. A median of 3066 and 2879 unique molecular identifiers (UMI) per cell for gemcitabine (GEM) Lin- and Control (Cont) Lin- were observed respectively. R [v4.1.0] and Python [v3.8.5] were used for all downstream analyses. Genes expressed in <10 cells were discarded. High-quality cells were retained by excluding: (i) cells expressing <1000 or >5000 unique genes and (ii) cells with a mitochondrial UMI proportion of >10% - yielding 13699 cells (GEM_Lin- = 7858, Cont_Lin- = 5841) and a total of 16007 detectable genes. *SCTransform* [v0.3.2] [36], accessed via *Seurat* [v4.0.3] [37], was utilized to normalize and scale the data, select 3000 variable features and linearly regress out any remaining influence of mitochondrial UMI% on downstream analyses. *SCTransform* specifically mitigates technical factors, but retains biological heterogeneity, improving downstream analysis. The raw data of the scRNA-seq is included in Table S2.

### Classification of cell types

To classify all 13699 cells in an unsupervised manner, *SingleR* [v1.6.1] [38] was utilized to compare the transcriptome of each cell to a dual-reference of sorted microarray (ImmGen) and mouse RNA-seq data provided by *celldex* [v1.2.0] [38]. 157 (1.14%) with ambiguous or poor-quality classifications were discarded – as determined by the SingleR *prunescores* function set to a threshold of 3 absolute mean deviations. Contaminating cells (i.e., Lin+ or CD45-) were discarded and classifications were broadly verified in a supervised manner using known hematopoietic stem cells (LSK) (*CD117,Sca1*), and progenitors (*CD34,IL7R,CD16/32*) marker genes.

### Dimensionality reduction, unsupervised clustering and differential abundance analysis

Data from all samples was aggregated and, as calculated by the Seurat [v4.0.3] functions RunPCA and RunUMAP (default parameters), the top 3000 variable features and 25 principal components were utilized to generate a uniform manifold approximation and projection (UMAP) for visualization of the data. To assess globular and cellular heterogeneity, transcriptionally distinct cell states were defined by shared k-nearest-neighbour (s-KNN) analysis and Louvain-Jaccard clustering via the Seurat [v4.0.3] functions FindNeighbors and FindClusters respectively, using a resolution of 0.75. Cellular neighborhoods displaying differential abundance between conditions were defined by DASeq [39] [v1.0.0] using the top 10 principal components and *k*-values of [50–1000] at 50 step-wise intervals. Non-significant neighborhoods were discarded, as determined by a random permutations test (*p* < 0.01).

### Data visualization

Gene expression and UMAPs were visualized using the Seurat [v4.0.3] function *DimPlot*. Where noted, *MAGIC* [v2.0.3] [40] was used to impute the data, based on an automatically calculated level of diffusion (parameter *t* = *auto*). Imputed data was solely used for the purposes of visualization.

### Cell cycle status

For each cell, cell cycle status was estimated as a position along a circular continuum using *Tricycle* [v1.0.0] [41], based on the abundance of known cell-cycle genes (e.g. Top3a).

### Differential gene expression analysis

All differentially expressed genes were identified using the scRNA-seq-specific tool *MAST* [v1.18.0] [42] accessed via the *Seurat* [v4.0.3] *FindMarkers* function. Significance was assessed by calculating adjusted FDR *p*-values using the Bonferroni correction method and a gene was considered to be differentially expressed if its log2 fold-change was > ± 0.35.

### Pathway analysis

To characterize differentially expressed genes, gene set enrichment analysis (GSEA) tests were performed using *clusterProfiler* [v4.0.0] [34] and gene-lists from the HALLMARK database [43] (biological processes) and *msigdbr* [v7.4.1] (category = C2, subcategory = REACTOME). Only significantly enriched (FDR < 0.01, Bonferroni correction method) processes and TFs were retained.

### Hematocrit measurements

Hematocrit measurements were performed in a clinical blood processing lab at the Rambam Healthcare Center, Haifa, Israel.

### Colony forming assay

For colony forming units (CFU) assay, red blood cells were lysed from bone marrow of naïve mice. Lin- cells were extracted and then seeded in triplicates at a concentration of 500 cells/well into 6-well culture plates with M3434 methylcellulose (Methocult, Stem Cell Technologies, Vancouver, Canada). Cells were incubated for 12 days. Plates were imaged with a ZEISS microscope and colonies were scored. The experiments were performed in 3 biological repeats.

### Statistical analysis

Data are expressed as mean ± SE. The statistical significance for the in vitro experiments was determined by either Student t-test for a comparison between two groups, or one way ANOVA for a comparison between multiple groups, followed by Tukey post-hoc statistical test, using GraphPad prism 5.0 software. For in vivo studies, *n* = 5 mice/group were used unless indicated otherwise. All mice were randomly grouped before treatment was initiated. Animals were excluded from the analysis if mice died during the experiment or demonstrated pathological conditions that are not related to their tumors. Differences between all groups were compared with each other, and statistical significance was set at *P* < 0.05.

## Supplementary information

Supplemental tables and figures

## Data Availability

Raw sequencing data and normalized expression matrices (scRNA-seq) will be provided upon request.
