# Supplementary material for: Chemotherapy-induced tumor immunogenicity is mediated in part by megakaryocyte-erythroid progenitors
Source: Oncogene. 2023 Jan 16;42(10):771–81. doi: 10.1038/s41388-023-02590-0 (PMC9984299; doi:10.1038/s41388-023-02590-0)
Supplement: Supplementary file 1 — Supplemental tables and figures [file 41388_2023_2590_MOESM1_ESM.pdf]

Supplemental Tables and Figures

Vorontsova et al.,

Table S1: Cell surface markers analyzed by flow cytometry

**Cell type**	**Surface markers**
HSPCs (Lin- cells)	Lin^-
Lin+ cells	Lin^+
LSK	Lin^- $Sca1^+$ $CD117^+$
CLP	Lin^- $Sca1^+$ $CD117^+$ $IL7R^+$
MEP	Lin^- $Sca1^-$ $CD117^+$ $Fc\gamma R^-$ $CD34^-$ or Lin^- $ERMAP^+$
CMP	Lin^- $Sca1^-$ $CD117^+$ $Fc\gamma R^-$ $CD34^+$
GMP	Lin^- $Sca1^-$ $CD117^+$ $Fc\gamma R^+$ $CD34^-$
CD8+ T cells	$CD8^+$
CD4+ T cells	$CD4^+$
Activated CD8+ T cells	$CD8^+$ $CD25^+$
NK cells	$NKp46^+$
Activated NK cells	$NKp46^+$ $CD107^+$
M-MDSC	$CD11b^+$ $Ly6C^+$ $Ly6G^-$
G-MDSC	$CD11b^+$ $Ly6C^{low}$ $Ly6G^-$
Proinflammatory macrophages	$CD11b^+$ $F4/80^+$ $CD11c^+$ $CD206^-$
Immunosuppressive macrophages	$CD11b^+$ $F4/80^+$ $CD11c^-$ $CD206^+$

Supplemental Figures

Figure S1

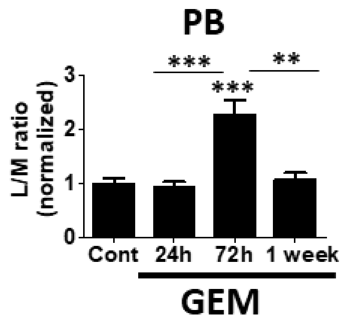

Figure S1: Lymphoid to myeloid ratio in peripheral blood is significantly elevated 72 hours following gemcitabine treatment. Panc02 cells ($5x10^5$ cells/mouse) were orthotopically implanted into the pancreas of C57BL/6 mice (n=5 mice/group). Three weeks later, treatment with gemcitabine (GEM, 500mg/kg) was initiated. After 24 h, 72 h or 1 week, peripheral blood (PB) was drawn and analyzed for lymphoid (CD3 and CD220) and myeloid (CD11b) cells by flow cytometry. Mice that were treated with a vehicle control were used as controls. Statistical significance was assessed by one-way ANOVA, followed by Tukey post-hoc test. Asterisks represent significance from control. Significant p values are shown as ** $p<0.01$; *** $p<0.001$.

Figure S2

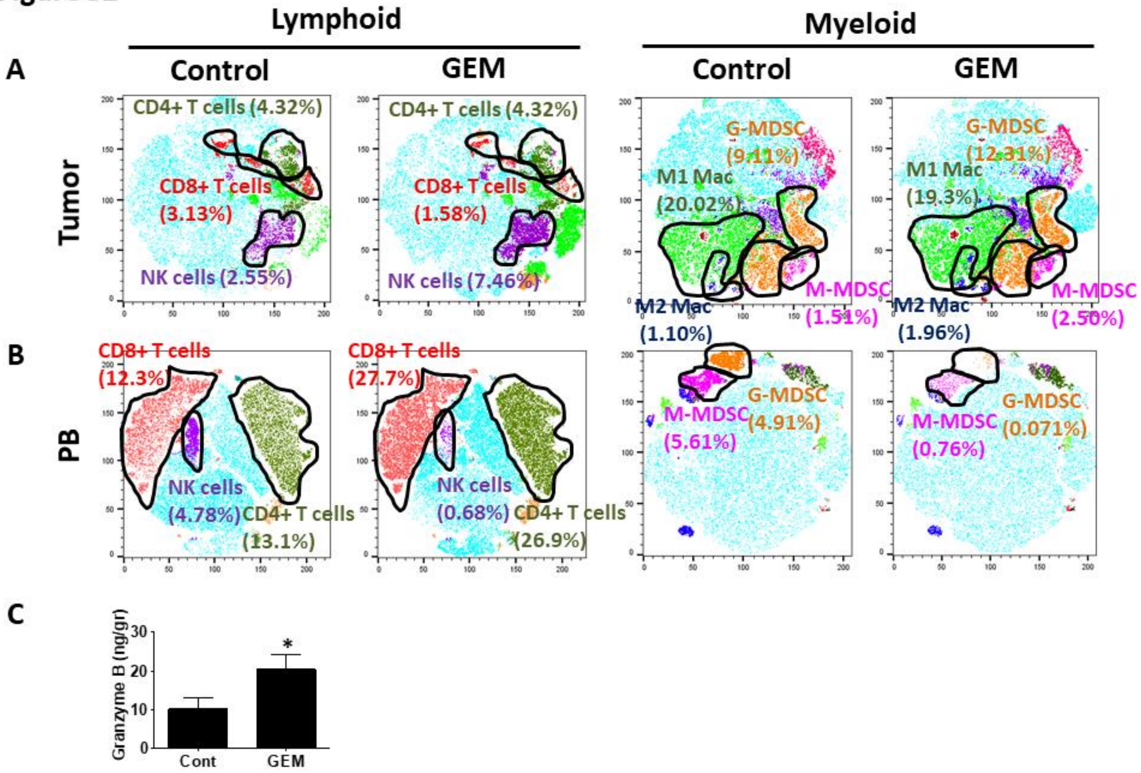

Figure S2: Immune cell composition in tumors and peripheral blood of gemcitabine-treated mice. Panc02 cells (5x10^{5} cells/mouse) were orthotopically implanted into the pancreas of C57BL/6 mice (n=7 mice/group). After 3 weeks, treatment with gemcitabine (GEM, 500mg/kg) was initiated. After 72 hours, tumors were harvested and blood was drawn. A-B. The percentages of lymphoid and myeloid cells were assessed in tumors (A) and peripheral blood (PB; B) by flow cytometry. Shown are tSNE plots. C. The levels of Granzyme B in tumor lysates were determined by ELISA. Statistical significance was assessed by unpaired one-tailed t-test. Significant p values are shown as *p<0.05..

Figure S3

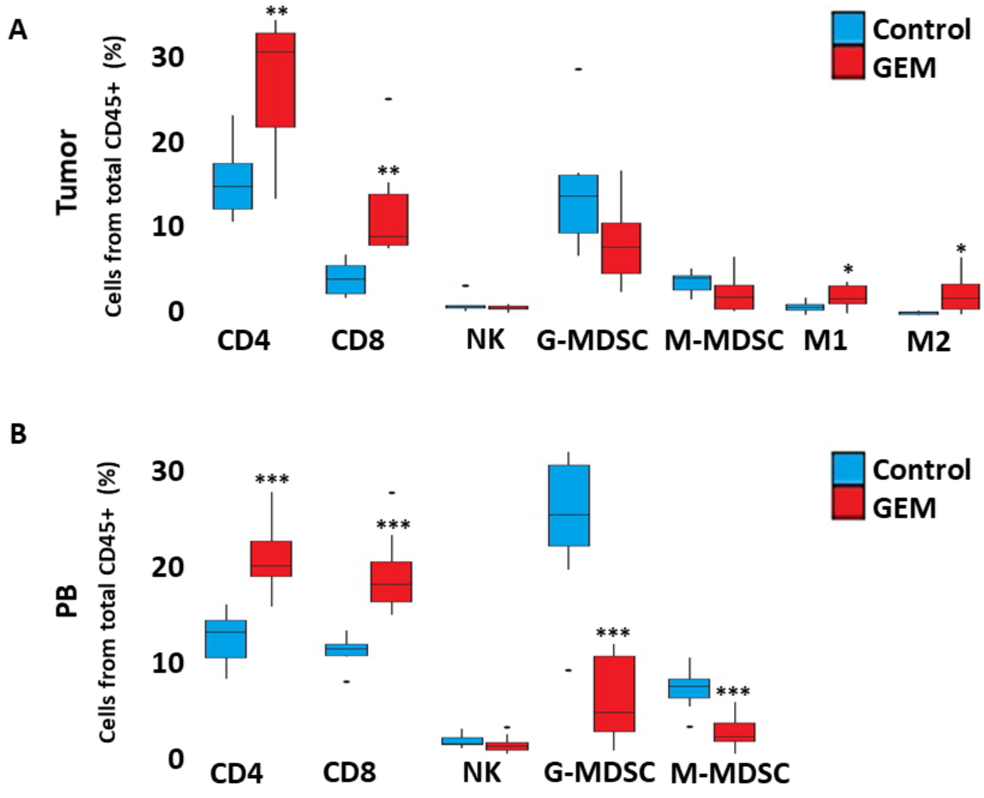

Figure S3: Immune cell composition in tumors and peripheral blood of mice treated with three cycles of gemcitabine. Panc02 cells (5x10^5 cells/mouse) were orthotopically implanted into the pancreas of C57BL/6 mice (n=7 mice/group). After three weeks, treatment with gemcitabine (GEM, 500mg/kg) was given once a week. Seventy-two hours after the third treatment, tumors were harvested and blood was drawn. A-B. Percentages of lymphoid and myeloid cells were assessed in tumors (A) and peripheral blood (PB; B) by flow cytometry. Statistical significance was assessed by unpaired one-tailed t-test. Significant p values are shown as *p<0.05; ** p<0.01; *** p<0.001.

Figure S4

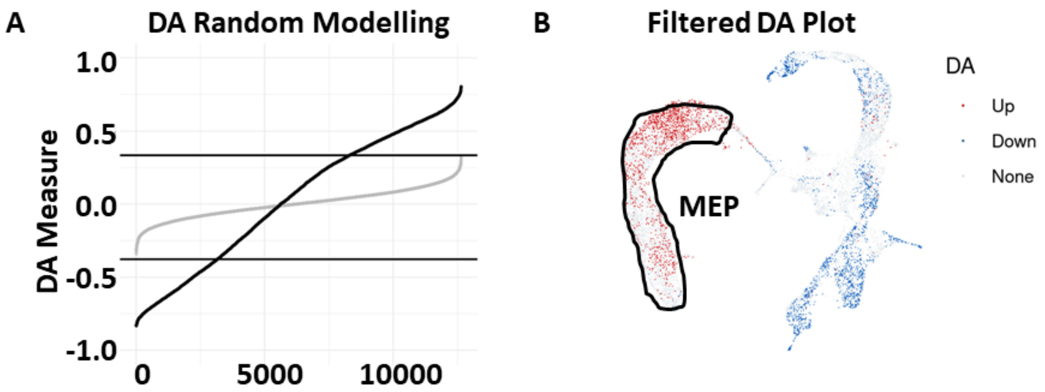

Figure S4: MEPs are significantly enriched in the bone marrow of mice treated with gemcitabine. A-B. Naïve, tumor-free mice were treated with GEM or vehicle control and sacrificed 72 h later. Lin- cells were obtained from the bone marrow and analyzed by single-cell RNA sequencing (scRNA-seq). A. Random modeling of differential abundance (DA) demonstrates a grey line representing DA scores obtained by chance compared with the black line representing the actual DA scores obtained from the scRNA-seq. A threshold is placed at the extremities of the random model and all DA scores above this are kept. B. Filtered DA plot demonstrates regions that exceed the threshold in A.

Figure S5

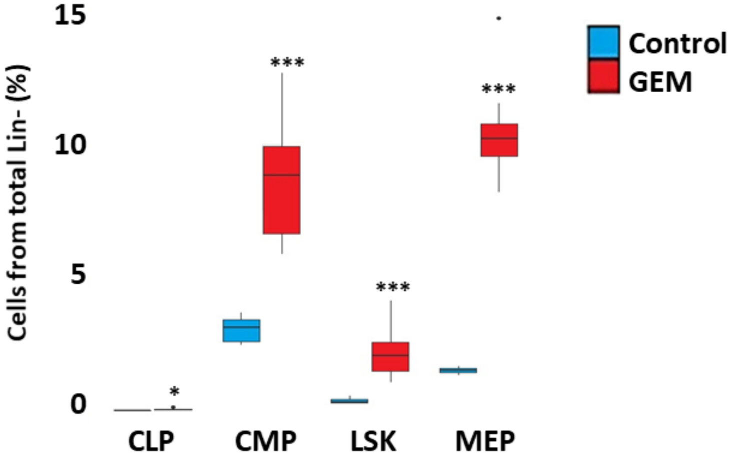

Figure S5: MEPs are enriched in the bone marrow of Panc02-bearing mice treated with three cycles of gemcitabine. Panc02 cells ($5x10^5$ cells/mouse) were orthotopically implanted into the pancreas of C57BL/6 mice (n=7 mice/group). After three weeks, treatment with gemcitabine (GEM, 500mg/kg) was given once a week. Seventy-two hours after the third treatment, the bone marrow was analyzed from HSPCs, and quantified by flow cytometry. Statistical significance was assessed by unpaired one-tailed t-test. Significant p values are shown as *p<0.05; *** p<0.001.

Figure S6

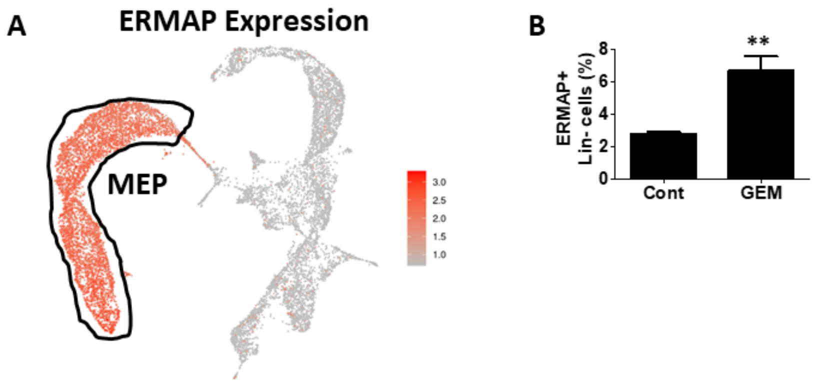

Figure S6: Megakaryocyte-erythroid progenitors exclusively express ERMAP. Tumor-free C57BL/6 mice were treated with gemcitabine (GEM) or vehicle control. Bone marrow cells were obtained 72 h later and analyzed by scRNA-seq and flow cytometry. A. ERMAP mRNA expression level in megakaryocyte-erythroid progenitors (MEPs) was determined from the scRNA-seq dataset. Shown is the UMAP plot of the overall dataset. B. Bone marrow cells were immunostained with lineage cocktail and ERMAP antibodies, and further gated on Lin- ERMAP+. Percentage of Lin- ERMAP+ cells (from total cells) is shown. Statistical significance was assessed by unpaired one-tailed t-test. Significant p values are shown as ** $p<0.01$.

Figure S7

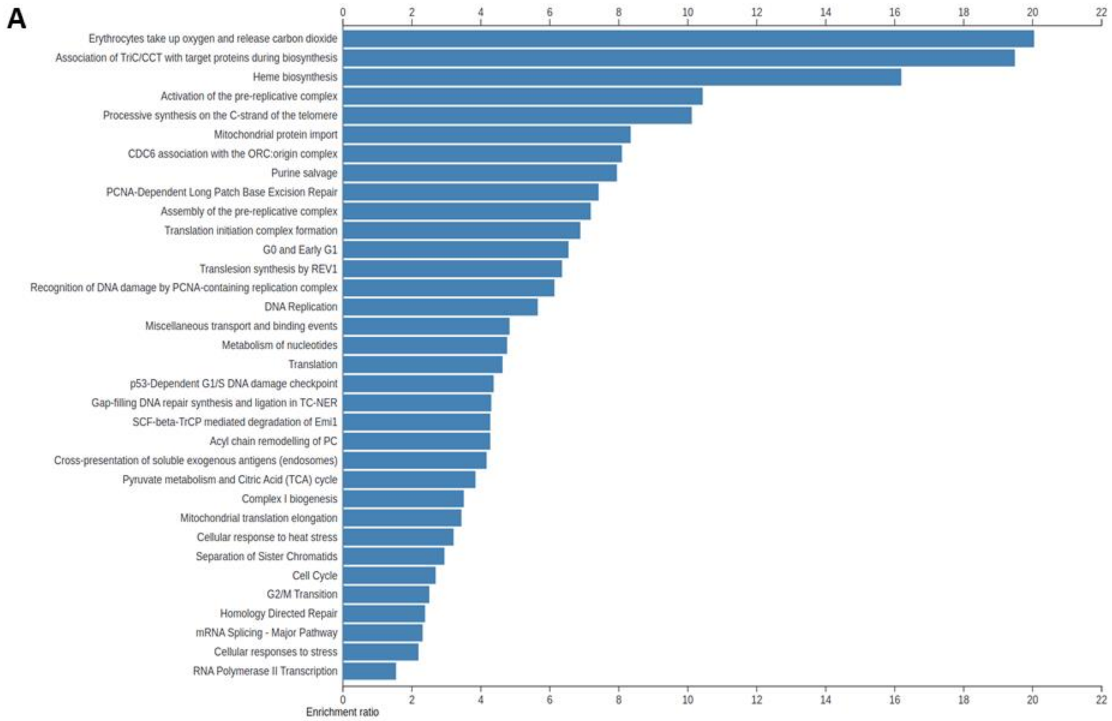

Figure S7: Pathways associated with oxygen species, heme and erythrocytes are enriched in megakaryocyte-erythroid progenitors. Analysis of mRNA levels of different genes from the scRNA-seq (from Figure 1), performed on Lin- cells. The differentially expressed genes of MEPs compared to all other Lin- cells, displayed as enrichment of biological pathways along with the enrichment ratio of each pathway.

Figure S8

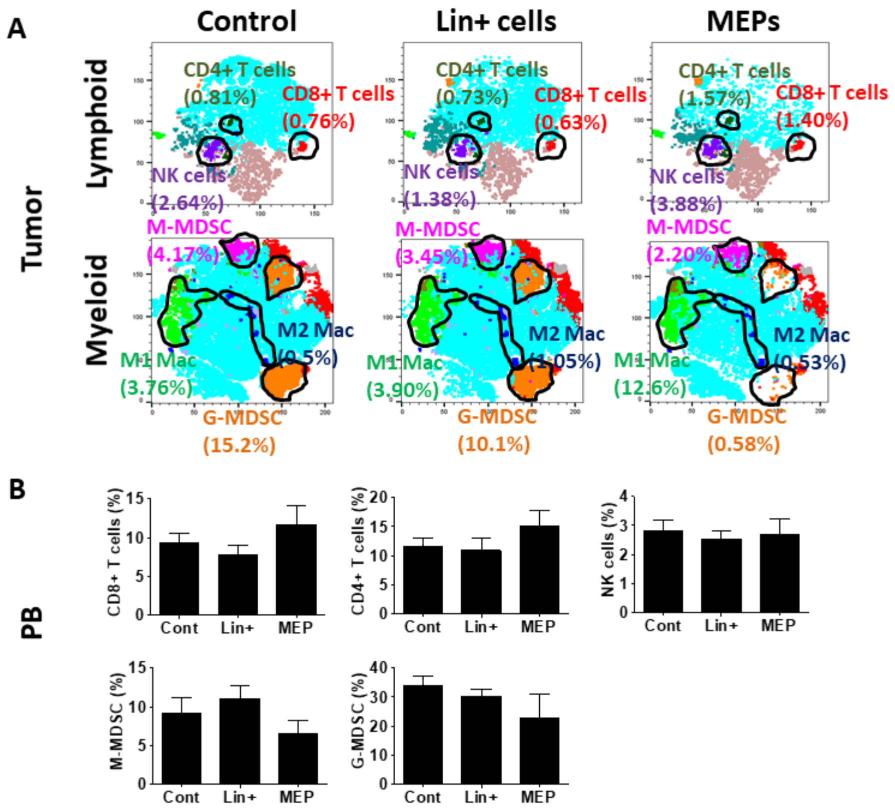

Figure S8: Immune cell composition in tumors and peripheral blood of mice adoptively transferred with megakaryocyte-erythroid progenitors. Megakaryocyte-erythroid progenitors (MEPs) or Lin+ cells were isolated from bone marrow of GFP+ mice. The GFP-tagged MEPs or Lin+ cells ($5x10^5$ cells/mouse) were injected every 5 days to mice bearing Panc02 tumors (n= 4-6 mice/group). Control mice were injected with saline. When control tumors reached endpoint, mice were sacrificed, and tumors and peripheral blood (PB) were harvested. The percentages of the indicated lymphoid and myeloid cells were assessed in tumors (A) and PB (B) by flow cytometry. tSNE plots are shown for tumors and graph bars are shown for peripheral blood. Percentages were calculated from CD45+ cells.

Figure S9

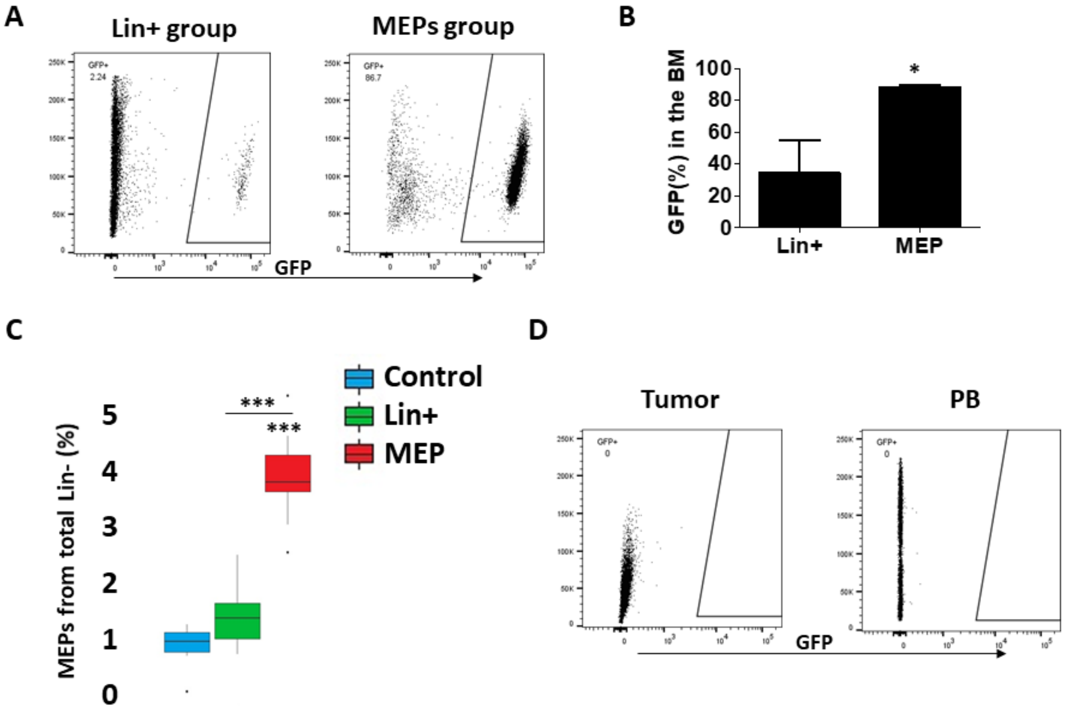

Figure S9: Megakaryocyte-erythroid progenitors home to the bone marrow upon adoptive transfer. Megakaryocyte-erythroid progenitors (MEPs) or Lin+ cells were isolated from bone marrow of GFP+ mice. The GFP-tagged MEPs or Lin+ cells ($5x10^5$ cells/mouse) were injected every 5 days to mice bearing Panc02 tumors (n= 4-6 mice/group). Control mice were injected with saline. A-B. The percentage of GFP+ cells in the bone marrow, representing the original transferred MEPs or Lin+ cells, and their differentiated cells was measured by flow cytometry at experiment end-point. Representative dotplots are shown (A), and bar plot is shown (B). C. The percentage of MEPs in the bone marrow of mice adoptively transferred with MEPs or Lin+ cells, as shown in Figure 3E, were analyzed by flow cytometry. D. The percentage of GFP+ cells in tumors and peripheral blood (PB) of mice adoptively transferred with MEPs was analyzed by flow cytometry. Representative dotplots are shown. Statistical significance was assessed by either one-way ANOVA, followed by Tukey post-hoc test, when comparison was made on more than 2 groups, or unpaired one-tailed t-test. Significant p values are shown as * $p<0.05$; *** $p<0.001$.

Figure S10

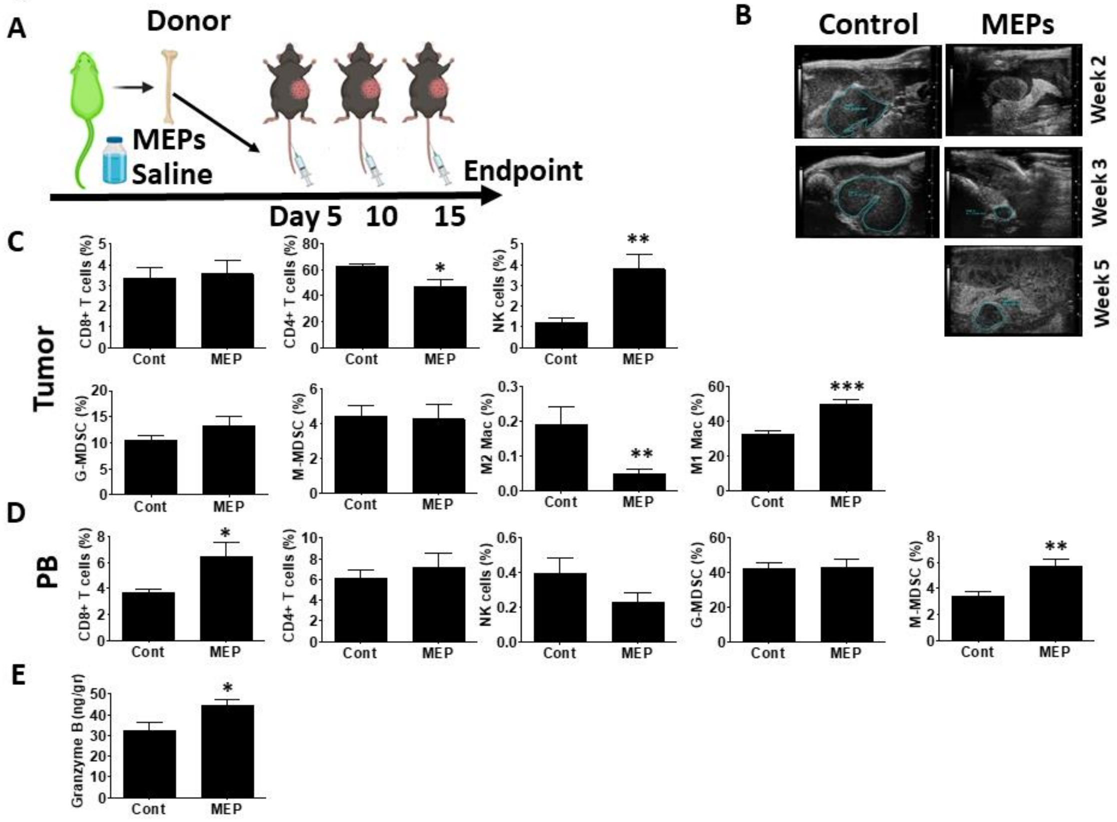

Figure S10: Megakaryocyte-erythroid progenitor adoptive transfer in a tumor size matched experiment. Megakaryocyte-erythroid progenitors (MEPs) were isolated from bone marrow of GFP+ mice. The GFP-tagged MEPs ($5x10^5$ cells/mouse) were injected every 5 days to mice bearing Panc02 tumors (n= 5-7 mice/group). Control mice were injected with saline. Tumor growth was monitored by micro-ultrasound (US). Mice were sacrificed when tumor size in each group reached an average size of $50mm^3$. A. A schematic representation of the adoptive transfer experiment is shown. B. Representative US images of tumors from each group over time. C-D. The percentages of the indicated lymphoid and myeloid cells were assessed in tumors (C) and peripheral blood (D) by flow cytometry. Percentages were calculated from CD45+ cells. E. The levels of Granzyme B in tumor lysates were determined by ELISA. Statistical significance was assessed by unpaired one-tailed t-test. Significant p values are shown as *p<0.05; ** p<0.01; *** p<0.001.

Figure S11

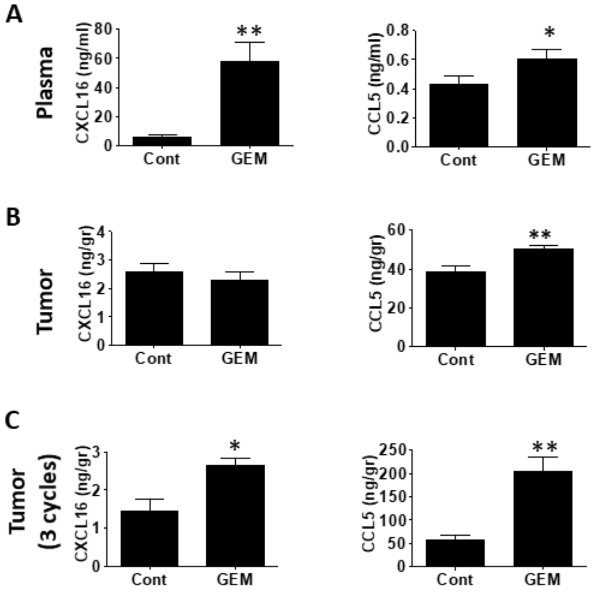

Figure 11: Levels of CXCL16 and CCL5 are increased in tumors and plasma from gemcitabine-treated mice. A-B. Panc02 cells ($5x10^5$ cells/mouse) were orthotopically implanted into the pancreas of C57BL/6 mice (n=4-5 mice/group). Three weeks later, mice were treated with gemcitabine (GEM, 500 mg/kg) or vehicle control. Blood was drawn and plasma was separated (A); and tumors were harvested and lysates were prepared (B). Samples were analyzed for CXCL16 and CCL5 levels using specific ELISAs. C. Panc02 cells ($5x10^5$ cells/mouse) were orthotopically implanted into the pancreas of C57BL/6 mice (n=7 mice/group). Treatment with gemcitabine (GEM, 500mg/kg) was given once a week. Seventy two hours after the third treatment, the tumors were harvested and prepared as lysates. Levels of CXCL16 and CCL5 were analyzed by specific ELISAs. Statistical significance was assessed by unpaired one-tailed t-test. Significant p values are shown as *p<0.05; ** p<0.01.
